# Evaluation of a partial optic nerve crush model in rats

**DOI:** 10.3892/etm.2012.619

**Published:** 2012-06-22

**Authors:** HAI-BO TAN, XI SHEN, YU CHENG, QIN JIAO, ZI-JIAN YANG, YI-SHENG ZHONG

**Affiliations:** Department of Ophthalmology, Ruijin Hospital Affiliated Medical School, Shanghai Jiaotong University, Shanghai 200025, P.R. China

**Keywords:** optic nerve, partial crush injury model, retinal ganglion cells

## Abstract

This study was performed to determine whether a partial optic nerve crush (PONC) model in rats is effective and reliable for the study of optic nerve protection and regeneration. Bilateral superior colliculus (SC) retrograde 1,1′-dioctadecyl-3,3,3′,3′-tetramethylindocarbocyanine perchlorate (DiI) labeling of retinal ganglion cells (RGCs; n=3) and unilateral SC retrograde labeling of RGCs (n=3) were performed in adult Sprague-Dawley (SD) rats and the results were compared with the bilateral and unilateral SC retrograde-labeled RGCs. Another 40 adult SD rats, three days after bilateral SC retrograde DiI labeling of RGCs underwent crushing with a non-invasive vascular clip (40 gram power) 1 mm behind the right optic nerve head for 5, 10 and 30 sec (n=10 each), and a sham-operated control group (n=10) was used as a control. The retinas of all 40 rats were flattened by four radial cuts, mounted vitreal side-up on gelatin-coated slides, and the number of labeled RGCs was counted in four distinct regions per retinal quadrant at three different eccentricities of 1/6, 3/6 and 5/6 of the retinal radius three days later. Bilateral SC retrograde DiI injection labeled the majority of normal RGCs, while unilateral SC injections only labeled a small part of the RGCs; the majority of RGCs were not labeled. In the mild crush (5 sec) injury group, the bilateral SC retrograde DiI injection labeled the majority of RGCs. The RGC densities at 1/6, 3/6 and 5/6 of the retinal radius showed no significant difference compared with the RGC densities at the corresponding region of the retinal radius in the sham-operated control group (P=0.734, 0.461, 0.273, respectively). In the moderate crush injury (10 sec) group, the number of labeled RGCs was significantly lower compared to that of the sham-operated control group, and the RGC densities at 1/6, 3/6, 5/6 of the retinal radius were significantly lower compared to the RGC densities at the corresponding retinal radius in the sham-operated control group (P<0.001). In the severe crush injury (30 sec) group the number of labeled RGCs was significantly decreased, and the labeled RGCs were not observed in the region at 5/6 of the retinal radius. The RGC densities at 1/6 and 3/6 of the retinal radius were significantly lower compared to the RGC densities at the corresponding retinal radius region in the sham-operated control group (P<0.001). Compared with the mild and severe optic nerve crush injury models, the moderate crush injury model is more suitable for the study of optic nerve damage and regeneration.

## Introduction

The optic nerve is a part of the central nervous system and is commonly used to study central nerve damage and regeneration due to its anatomical particularity. Approximately 95% of rat retinal ganglion cell (RGC) fibers project to the superior colliculus (SC) and can be labeled via injection of a fluorescent tracer into the rat bilateral SC (1,2). Retrograde labeling of RGCs with 1,1′-dioctadecyl-3,3,3′,3′-tetramethylindocarbocyanine perchlorate (DiI) and examining the retinas using fluorescence microscopy following periods of survival, is effective and reliable for the observation of dynamic changes in RGCs.

An optic nerve lesion not only causes typical morphological change but is able to activate signal transduction which may reduce neuronal loss and promote intrinsic axonal regeneration (3). Partial optic nerve crush (PONC), an experimental procedure of a standardized and reproducible incomplete axotomy of the RGCs, mimics the key pathological progress which is related to RGC apoptosis (4). In the present study, we evaluated the reliability of the PONC model by retrograde labeling of RGCs by DiI.

## Materials and methods

### Animals

All experiments were performed in compliance with guidelines for animal care of the Association for Research in Vision and Ophthalmology. A total of 46 adult male Sprague-Dawley (SD) rats with a body weight of 150–200 g were used in the study. During experimentation, animals were housed under a 12:12-h light/dark cycle, with water and food available *ad libitum*.

### SC retrograde DiI labeling

The DiI suspension was prepared by mixing 3 mg DiI in 1 ml saline containing 1–3% Triton X-100. Sonication and repeated agitation produced a mixture of dissolved DiI and small DiI crystals in suspension. The animals were anesthetized via intraperitoneal injection of 20% chloral hydrate (420 mg/kg) prior to surgery and were fixed in a stereotaxic apparatus. Following skin removal, the cranium was exposed and pierced by a 50-ml injector needle. The position of the SC was located at the point behind the fonticuli minor 6.4 mm apart from the mid-line 1.5 mm and inserting needle 4.0 mm. Using a micro-injector, 1.5 μl 10% DiI was injected at each point. To compare the effect of the bilateral and unilateral SC retrograde labeling, six rats were divided into two groups at random (three rats per group). Another 40 rats with bilateral SC retrograde labeling underwent partial optic nerve crush.

### PONC model

The animals were anesthetized via intraperitoneal injection of 20% chloral hydrate (420 mg/kg) after SC retrograde DiI labeling for three days. The optic nerve of the right eye in all groups was exposed by opening the meninges of the optic nerve with the sharp tips of forceps, followed by blunt dissection. The exposed optic nerve was then partially crushed 1 mm behind the globe for 5, 10 and 30 sec (mild, moderate and severe crush, respectively) each with 40 gram power (n=10). A sham-operated control group (n=10) was treated in the same way on the right eye, but without closing of the the forceps, to check for any falsifying influence of surgery on the treatment effects. In all cases, the retinal blood supply remained grossly intact, as judged on the basis of a direct microscopic inspection during and after the procedure.

### Quantification of RGCs

The rats were anesthetized via intraperitoneal injection of 20% chloral hydrate (420 mg/kg) following DiI application for six days and perfused transcardially with saline and 4% paraformaldehyde (PFA) for 30 min. Following enucleation, the eyes were postfixed for 1 h in 4% PFA solution. The retinas were dissected, vitreal side-up flat-mounted on gelatin-coated slides and RGC counts were performed immediately using laser confocal fluorescence microscope. The cell count was performed in an area approximately the same distance from the optic disc; 1/6, 3/6 and 5/6 of the retinal radius. Three fields were selected where images were obtained using a digital imaging system (ImagePro 6.0) and the average number of RGCs/mm^2^ was calculated.

### Statistics

Data were analyzed using SPSS 19.0 software (SPSS, Chicago, IL, USA). Data were tested for statistical significance with the independent samples t-test or by analysis of variance (one-way ANOVA). A P-value <0.05 was considered to indicate a statistically significant result.

## Results

### SC retrograde DiI-labeled normal RGCs

**Figure 1:**
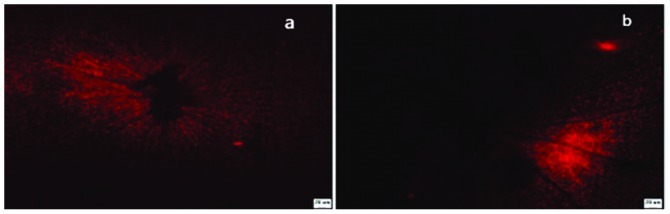
(a) Bilateral SC retrograde DiI-labeled normal RGCs, (b) unilateral SC retrograde DiI-labeled normal RGCs. SC, superior colliculus; RGCs, retinal ganglion cells; DiI, 1,1′-dioctadecyl-3,3,3′,3′-tetramethylindocarbocyanine perchlorate.

The majority of RGCs were labeled with bilateral SC retrograde DiI injection and there were more RGCs adjacent to the optic nerve area than compared with the number in the retina perimeter; no RGCs were labeled in the blood vessel area (Fig. 1a). The majority of RGCs were not labeled following unilateral SC retrograde DiI injection (Fig. 1b).

### Bilateral SC retrograde DiI-labeled RGCs in PONC model

**Figure 2:**
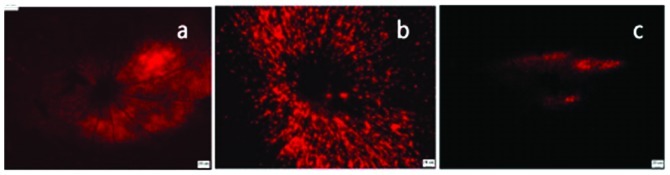
Bilateral SC retrograde DiI-labeled RGCs of various PONC extents: (a) 5 sec group (mild), (b) 10 sec group (moderate) and (c) 30 sec (severe) group. Bar, 20 μm. SC, superior colliculus; RGCs, retinal ganglion cells; PONC, partial optic nerve crush; DiI, 1,1′-dioctadecyl-3,3,3′,3′-tetramethylindocarbocyanine perchlorate.

**Figure 3:**
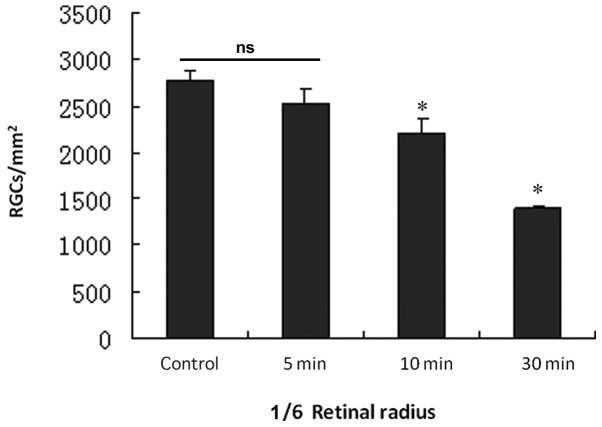
RGC densities as a result of the various PONC processes (1/6 retinal radius). ^*^P<0.001 compared with normal group; ns, no significance. RGCs, retinal ganglion cells; PONC, partial optical nerve crush.

**Figure 4:**
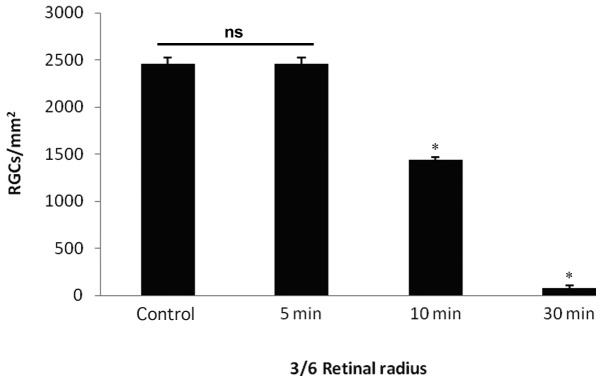
RGC densities as a result of the various PONC processes (3/6 retinal radius). ^*^P-values <0.001 compared with normal group; ns, no significance; RGCs, retinal ganglion cells; PONC, partial optical nerve crush.

**Figure 5:**
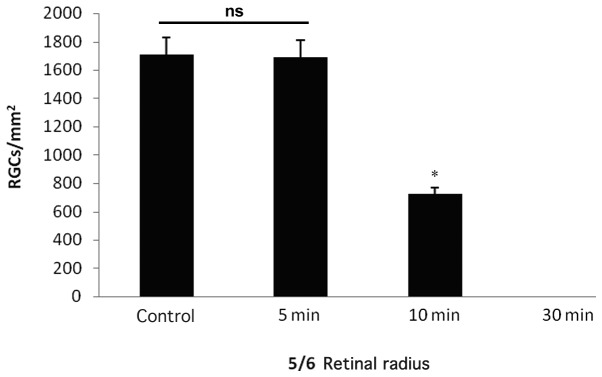
RGC densities as a result of the various PONC processes (5/6 retinal radius). ^*^P<0.001 compared with normal group; ns, no significance; RGCs, retinal ganglion cells; PONC, partial optical nerve crush.

The effects of the various PONC extents (5, 10 and 30 sec) on the numbers of labeled RGCs are shown in Figs. 2–5 and Table I. In the 5 sec PONC group the majority of RGCs were labeled (Fig. 2a). The RGC densities in the regions 1/6, 3/6 and 5/6 of the retinal radius compared to the sham-operated control group were not significantly different (P-values = 0.734, 0.461, 0.273; Figs. 3–5 and Table I). In the 10 sec PONC group, the numbers of labeled RGCs were less than that in the sham-operated control group (Fig. 2b), however, the 10-sec procedure led to a significant decrease in RGC numbers in regions 1/6, 3/6 and 5/6 of the retinal radius compared to RGC numbers in the sham-operated control group (P-values = 0.000, 0.000, 0.000; Figs. 3–5 and Table I). In the 30 sec PONC group, the number of labeled RGCs were significantly decreased (Fig. 2c); some cells in the region 1/6 retinal of the radius were labeled. However, fewer cells in the region 3/6 of the retinal radius were labeled and almost no cells were labeled in the region 5/6 of the retinal radius. The 30 sec PONC led to a significant decrease in RGCs in 1/6 and 3/6 retinal radius regions compared to these values in the the sham-operated control group (P-values = 0.000, 0.000; Figs. 3–4 and Table I).

## Discussion

Fluorescent substances, including DiI and Fluorogold, are able to transfer from the SC or lateral geniculate body or optic nerve stump to RGCs and obtain a fluorescent effect due to retrograde axoplasmic transport. DiI was used as a tracer due to its capacity to diffuse within the plasmalemma and label RGCs, while the other types of cells are not stained. Other tracers, including fast blue and nuclear yellow, diffuse easily to other cells and are difficult to preserve. Using the traditional histochemical technology it is difficult to distinguish RGCs from other cells, especially amacrine cells. Compared with the fast blue and nuclear yellow tracers, bilateral SC retrograde DiI-labeling is a reliable and effective method to study the dynamic pathological change of RGCs, and the method is already widely used in the fields of RGC apoptosis and optic nerve regeneration (5).

As 95% of RGCs project to the bilateral SC and only approximately 10% project to the unilateral SC, it is not reliable to label RGCs via unilateral SC injection since at least 10% of RGCs will not be labeled (1). The trilateral injection is more reliable, however, it requires a longer surgical time and may increase the risk of infection. The present study found that the bilateral SC retrograde DiI injection uniformly labeled the RGCs, that the number of RGCs among the different quadrants was not statistically different and that the number of RGCs from the center compared with the peripheral retina was not significantly attenuated. Thus, we suggest that the bilateral SC retrograde DiI injection is more efficient, improves labeling of RGCs and is more stable.

The PONC model is widely used in the fields concerned with RGC protection and optic nerve regeneration. The present study found that the majority of RGCs were labeled in the 5 sec PONC group (Fig. 2a) and that the RGC densities in 1/6, 3/6 and 5/6 retinal radius regions compared with the sham-operated control group were not significantly different, however, the number of labeled RGCs significantly decreased and almost no cells were labeled in the 5/6 retinal radius area in the 30 sec PONC group. In the 10 sec PONC group, the number of labeled RGCs was less than that in the control group, and the process led to a significant decrease in RGCs in 1/6, 3/6 and 5/6 retinal radius regions compared to these numbers in the sham-operated control group, which was similar to the findings of a previous study (6). Compared with the mild and severe optic nerve crush injury model, the moderate crush injury model (10 sec PONC group) met the demands of the PONC model and was more suitable for the study of optic nerve damage and regeneration.

## Figures and Tables

**Table I:** RGC densities as a result of different PONC processes (n=10, mean ± SD).

	RGCs density (cells/mm^2^)		
Group	1/6 Retinal radius	3/6 Retinal radius	5/6 Retinal radius
Normal group	2779.80±96.45	2457.75±63.76	1709.00±119.49
5 sec group	2522.60±159.74	2455.75±65.05	1692.00±118.67
10 sec group	2210.00±156.17^a^	1438.75±30.96^a^	722.60±44.34^a^
30 sec group	1393.00±20.94^a^	80.60±21.31^a^	0
F	5.42	12.21	35.04
P-value	0.000	0.000	0.000

a P<0.001 compared with the normal group. F, F-value (ANOVA); RGCs, retinal ganglion cells; PONC, partial optical nerve crush; SD, standard deviation.
